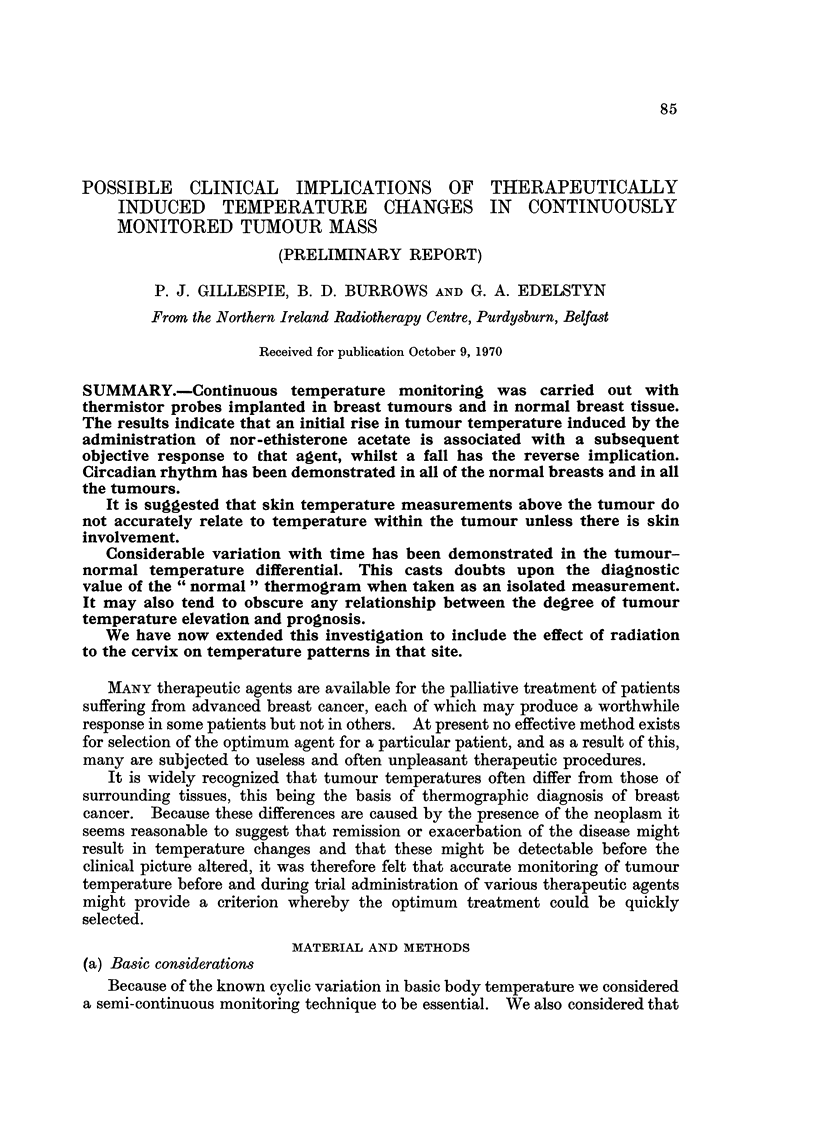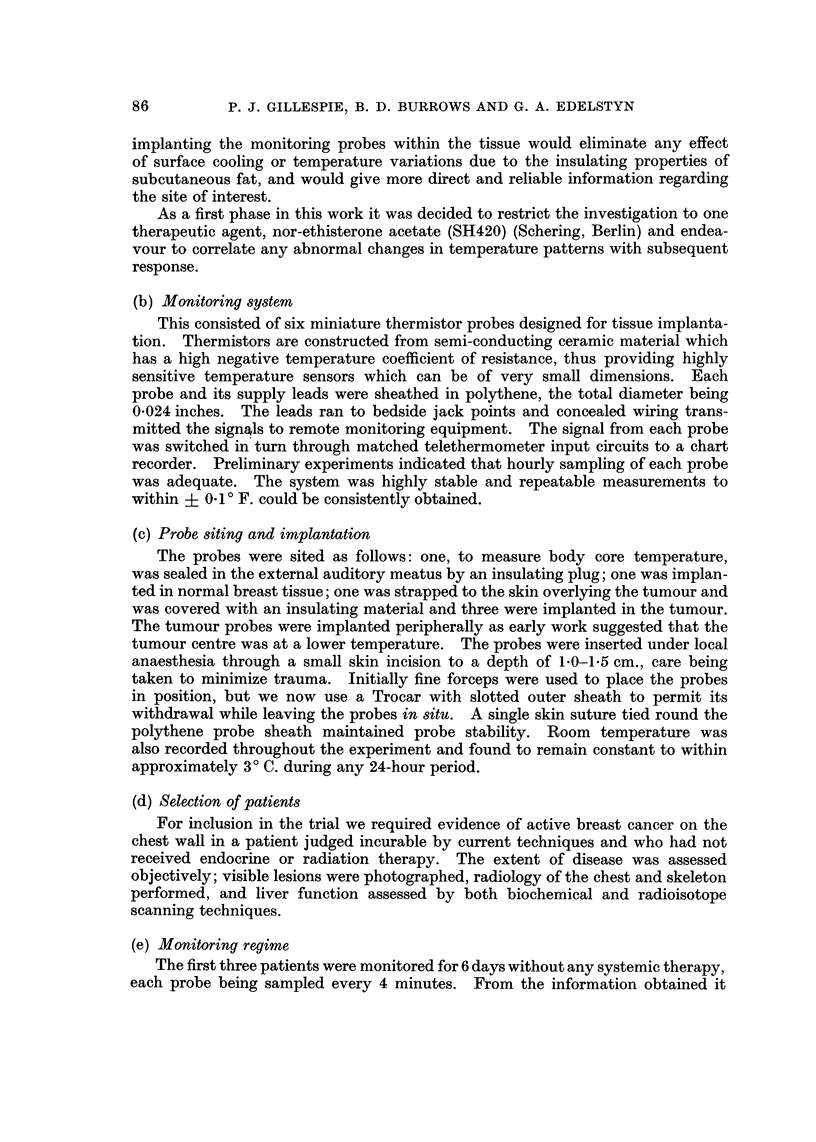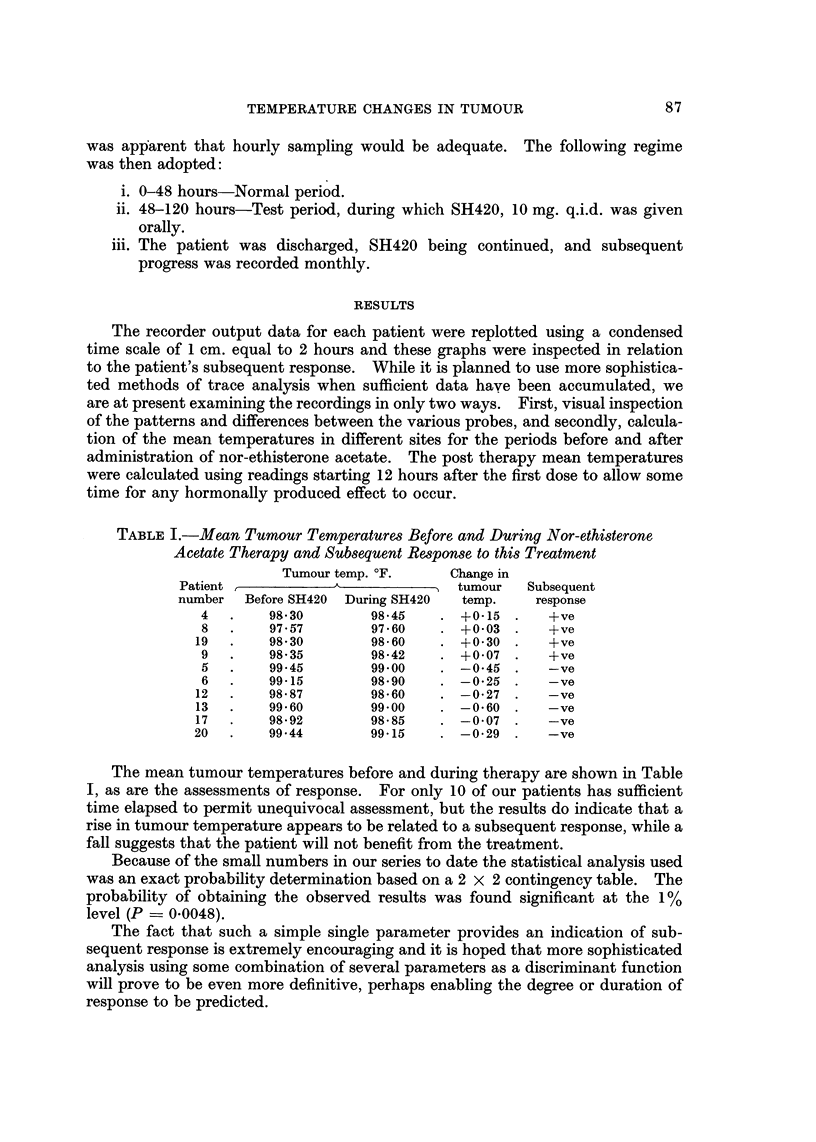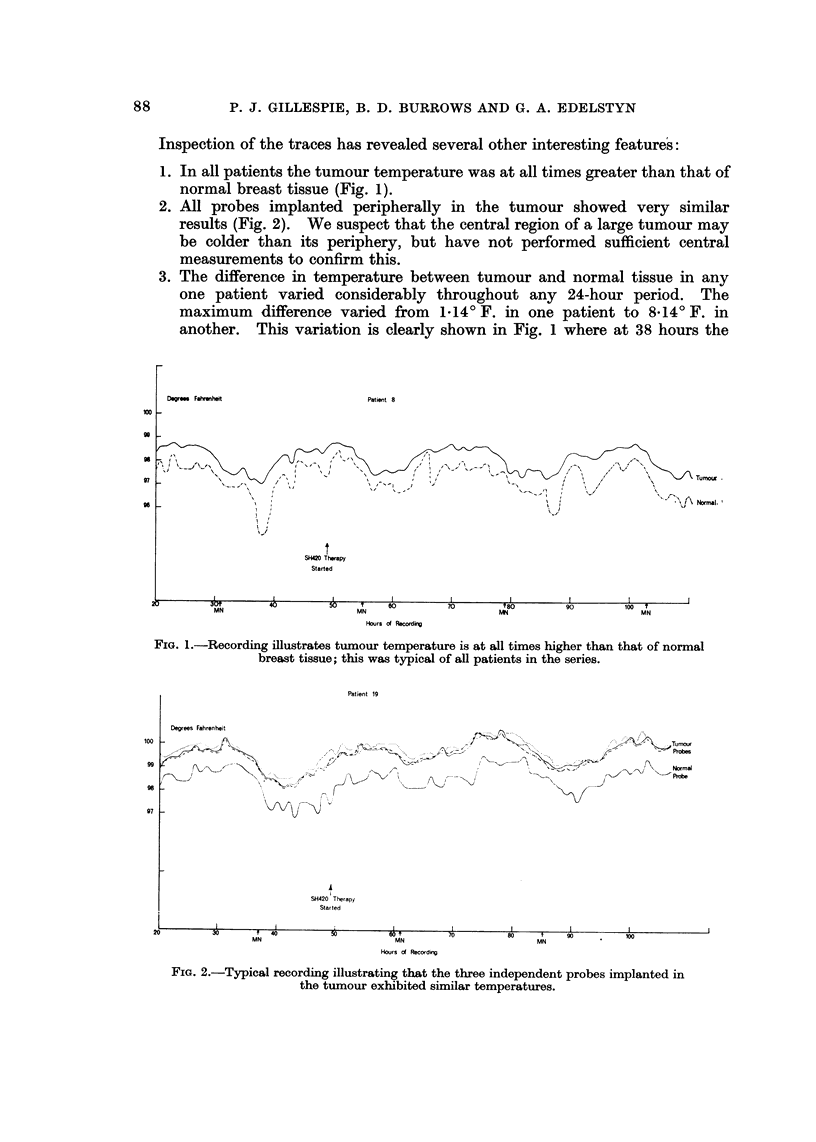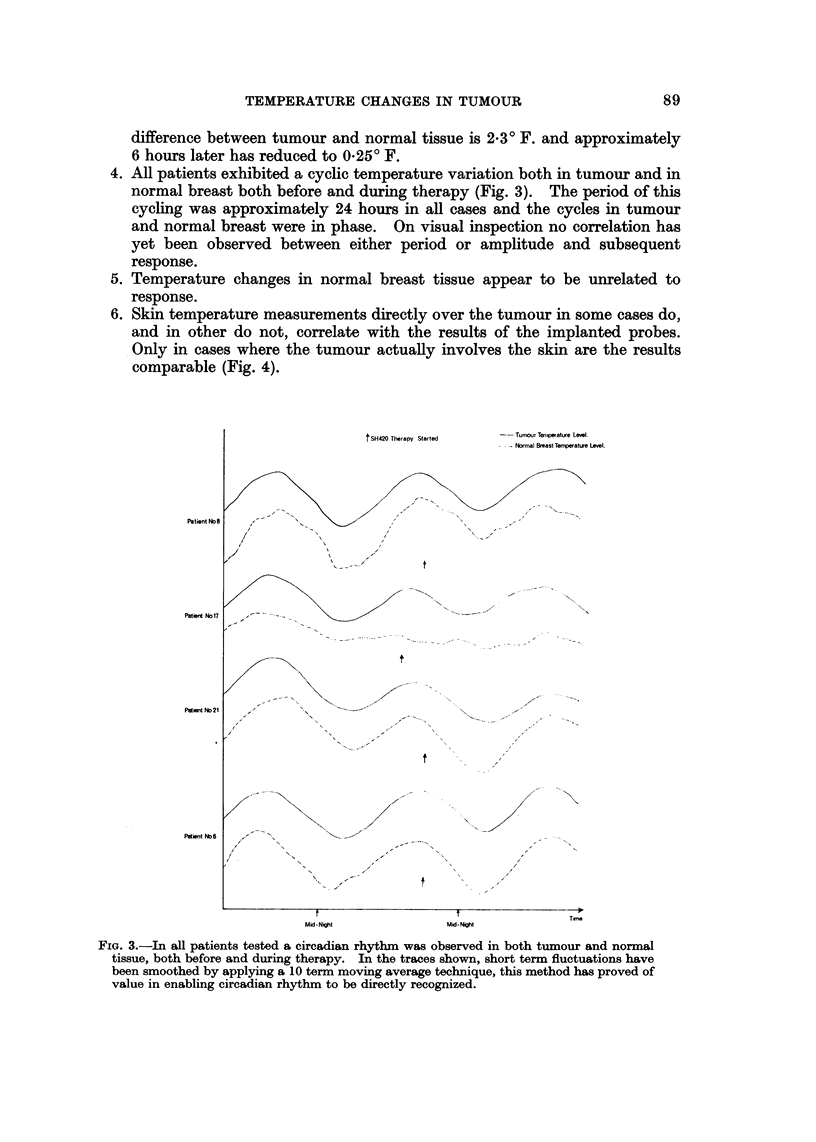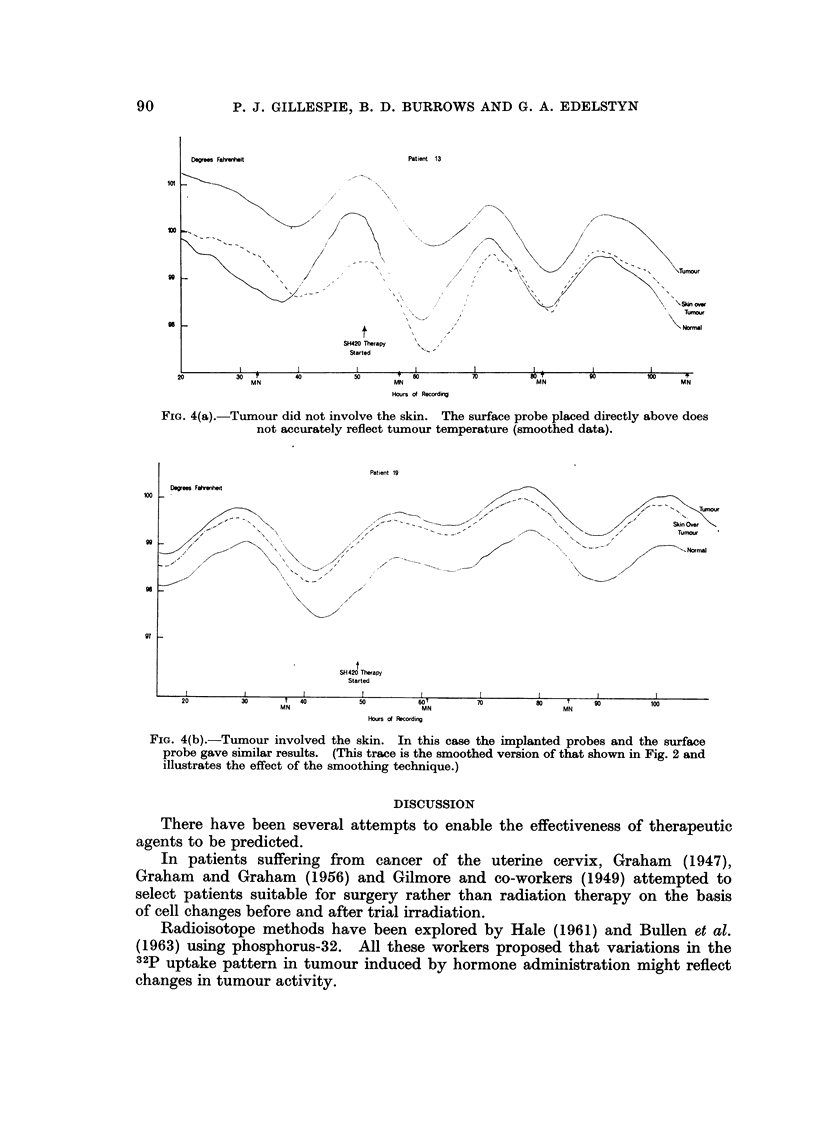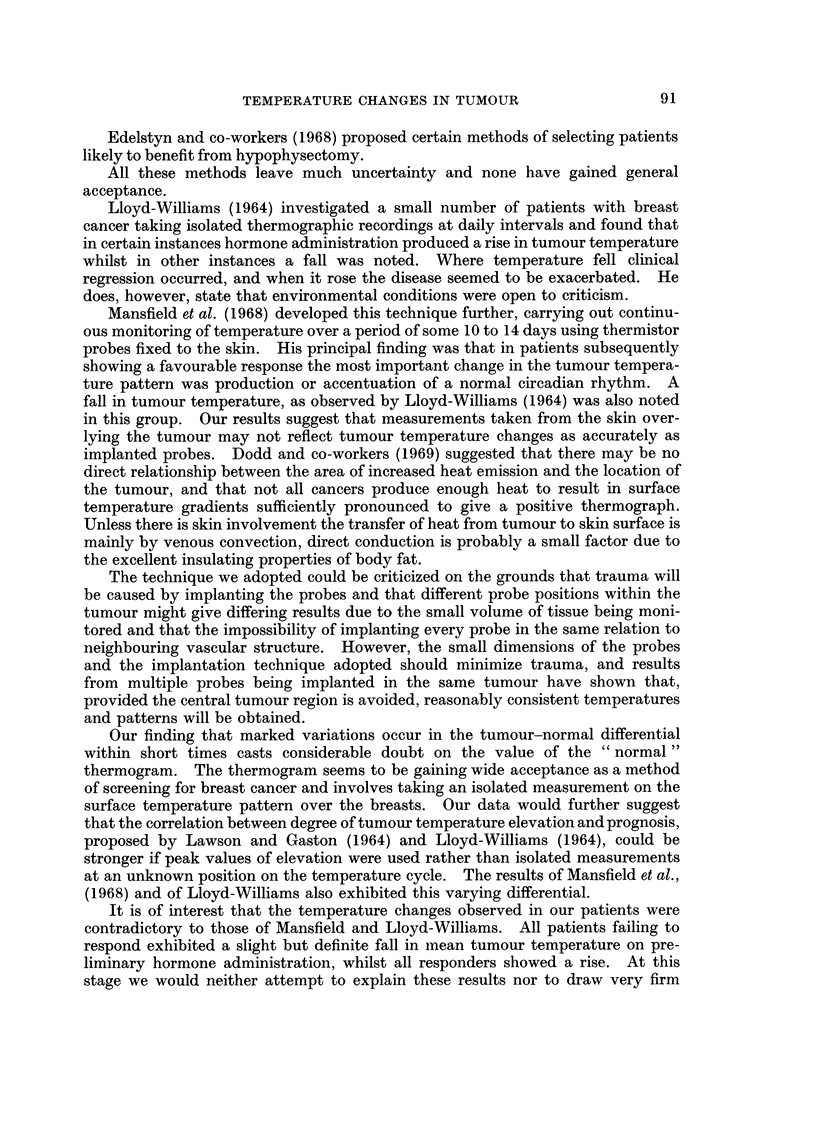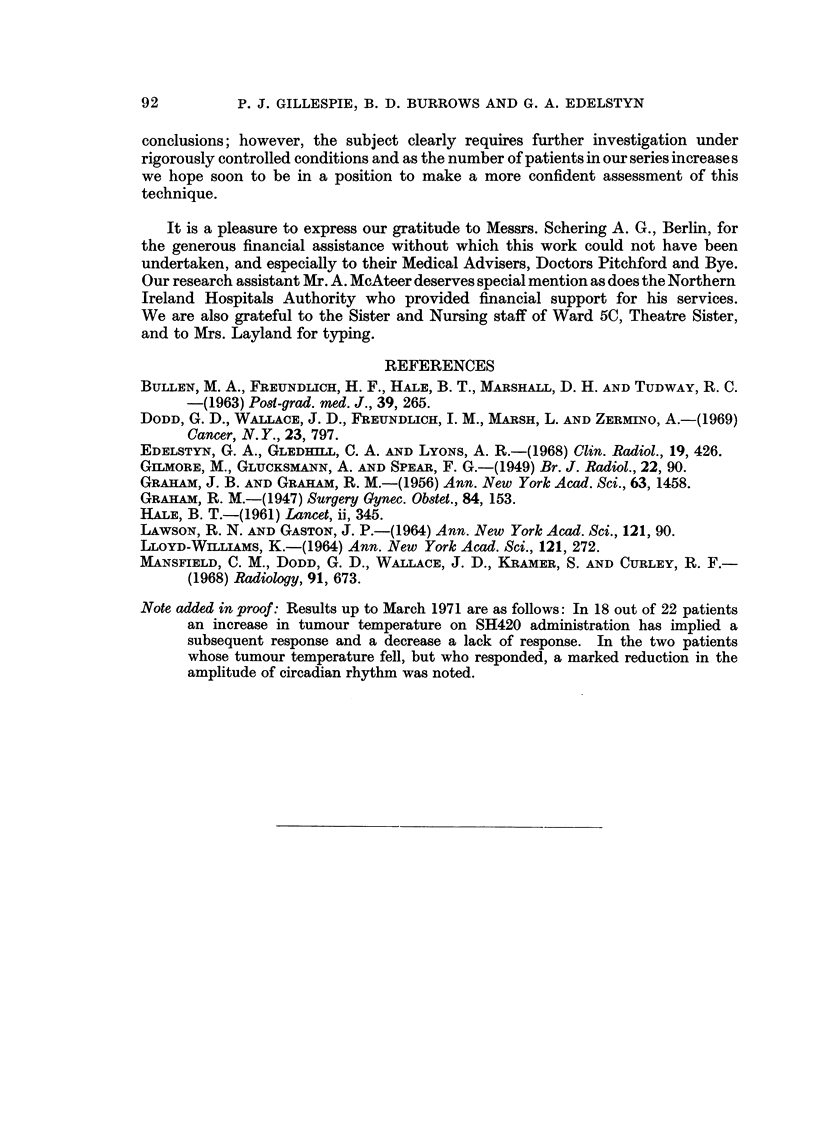# Possible Clinical Implications of Therapeutically Induced Temperature Changes in Continuously Monitored Tumour mass

**DOI:** 10.1038/bjc.1971.12

**Published:** 1971-03

**Authors:** P. J. Gillespie, B. D. Burrows, G. A. Edelstyn

## Abstract

Continuous temperature monitoring was carried out with thermistor probes implanted in breast tumours and in normal breast tissue. The results indicate that an initial rise in tumour temperature induced by the administration of nor-ethisterone acetate is associated with a subsequent objective response to that agent, whilst a fall has the reverse implication. Circadian rhythm has been demonstrated in all of the normal breasts and in all the tumours.

It is suggested that skin temperature measurements above the tumour do not accurately relate to temperature within the tumour unless there is skin involvement.

Considerable variation with time has been demonstrated in the tumour-normal temperature differential. This casts doubts upon the diagnostic value of the “normal” thermogram when taken as an isolated measurement. It may also tend to obscure any relationship between the degree of tumour temperature elevation and prognosis.

We have now extended this investigation to include the effect of radiation to the cervix on temperature patterns in that site.


### POSSIBLE CLINICAL IMPLICATIONS OF THERAPEUTICALLY INDUCED TEMPERATURE CHANGES IN CONTINUOUSLY MONITORED TUMOUR MASS

### (PRELIMINARY REPORT)

P. J. GILLESPIE, B. D. BURROWS AND G. A. EDELSTYN

*From the Northern Ireland Radiotherapy Centre, Purdysburn, Belfast*

Received for publication October 9, 1970

**SUMMARY.—Continuous temperature monitoring was carried out with thermistor probes implanted in breast tumours and in normal breast tissue. The results indicate that an initial rise in tumour temperature induced by the administration of nor-ethisterone acetate is associated with a subsequent objective response to that agent, whilst a fall has the reverse implication. Circadian rhythm has been demonstrated in all of the normal breasts and in all the tumours.**

**It is suggested that skin temperature measurements above the tumour do not accurately relate to temperature within the tumour unless there is skin involvement.**

**Considerable variation with time has been demonstrated in the tumour–normal temperature differential. This casts doubts upon the diagnostic value of the " normal " thermogram when taken as an isolated measurement. It may also tend to obscure any relationship between the degree of tumour temperature elevation and prognosis.**

**We have now extended this investigation to include the effect of radiation to the cervix on temperature patterns in that site.**

MANY therapeutic agents are available for the palliative treatment of patients suffering from advanced breast cancer, each of which may produce a worthwhile response in some patients but not in others. At present no effective method exists for selection of the optimum agent for a particular patient, and as a result of this, many are subjected to useless and often unpleasant therapeutic procedures.

It is widely recognized that tumour temperatures often differ from those of surrounding tissues, this being the basis of thermographic diagnosis of breast cancer. Because these differences are caused by the presence of the neoplasm it seems reasonable to suggest that remission or exacerbation of the disease might result in temperature changes and that these might be detectable before the clinical picture altered, it was therefore felt that accurate monitoring of tumour temperature before and during trial administration of various therapeutic agents might provide a criterion whereby the optimum treatment could be quickly selected.

### MATERIAL AND METHODS

### (a) *Basic considerations*

Because of the known cyclic variation in basic body temperature we considered a semi-continuous monitoring technique to be essential. We also considered that implanting the monitoring probes within the tissue would eliminate any effect of surface cooling or temperature variations due to the insulating properties of subcutaneous fat, and would give more direct and reliable information regarding the site of interest.

As a first phase in this work it was decided to restrict the investigation to one therapeutic agent, nor-ethisterone acetate (SH420) (Schering, Berlin) and endeavour to correlate any abnormal changes in temperature patterns with subsequent response.

(b) *Monitoring system*

This consisted of six miniature thermistor probes designed for tissue implantation. Thermistors are constructed from semi-conducting ceramic material which has a high negative temperature coefficient of resistance, thus providing highly sensitive temperature sensors which can be of very small dimensions. Each probe and its supply leads were sheathed in polythene, the total diameter being 0·024 inches. The leads ran to bedside jack points and concealed wiring transmitted the signals to remote monitoring equipment. The signal from each probe was switched in turn through matched telethermometer input circuits to a chart recorder. Preliminary experiments indicated that hourly sampling of each probe was adequate. The system was highly stable and repeatable measurements to within $\pm$ 0·1° F. could be consistently obtained.

(c) *Probe siting and implantation*

The probes were sited as follows: one, to measure body core temperature, was sealed in the external auditory meatus by an insulating plug; one was implanted in normal breast tissue; one was strapped to the skin overlying the tumour and was covered with an insulating material and three were implanted in the tumour. The tumour probes were implanted peripherally as early work suggested that the tumour centre was at a lower temperature. The probes were inserted under local anaesthesia through a small skin incision to a depth of 1·0–1·5 cm., care being taken to minimize trauma. Initially fine forceps were used to place the probes in position, but we now use a Trocar with slotted outer sheath to permit its withdrawal while leaving the probes *in situ*. A single skin suture tied round the polythene probe sheath maintained probe stability. Room temperature was also recorded throughout the experiment and found to remain constant to within approximately 3° C. during any 24-hour period.

(d) *Selection of patients*

For inclusion in the trial we required evidence of active breast cancer on the chest wall in a patient judged incurable by current techniques and who had not received endocrine or radiation therapy. The extent of disease was assessed objectively; visible lesions were photographed, radiology of the chest and skeleton performed, and liver function assessed by both biochemical and radioisotope scanning techniques.

(e) *Monitoring regime*

The first three patients were monitored for 6 days without any systemic therapy, each probe being sampled every 4 minutes. From the information obtained it was apparent that hourly sampling would be adequate. The following regime was then adopted:

i. 0–48 hours—Normal period.
ii. 48–120 hours—Test period, during which SH420, 10 mg. q.i.d. was given orally.
iii. The patient was discharged, SH420 being continued, and subsequent progress was recorded monthly.

### RESULTS

The recorder output data for each patient were replotted using a condensed time scale of 1 cm. equal to 2 hours and these graphs were inspected in relation to the patient's subsequent response. While it is planned to use more sophisticated methods of trace analysis when sufficient data have been accumulated, we are at present examining the recordings in only two ways. First, visual inspection of the patterns and differences between the various probes, and secondly, calculation of the mean temperatures in different sites for the periods before and after administration of nor-ethisterone acetate. The post therapy mean temperatures were calculated using readings starting 12 hours after the first dose to allow some time for any hormonally produced effect to occur.

TABLE I.—*Mean Tumour Temperatures Before and During Nor-ethisterone Acetate Therapy and Subsequent Response to this Treatment*

| Patient number | Tumour temp. °F. Before SH420 | During SH420 | Change in tumour temp. | Subsequent response |
|---|---|---|---|---|
| 4 | 98·30 | 98·45 | +0·15 | +ve |
| 8 | 97·57 | 97·60 | +0·03 | +ve |
| 19 | 98·30 | 98·60 | +0·30 | +ve |
| 9 | 98·35 | 98·42 | +0·07 | +ve |
| 5 | 99·45 | 99·00 | −0·45 | −ve |
| 6 | 99·15 | 98·90 | −0·25 | −ve |
| 12 | 98·87 | 98·60 | −0·27 | −ve |
| 13 | 99·60 | 99·00 | −0·60 | −ve |
| 17 | 98·92 | 98·85 | −0·07 | −ve |
| 20 | 99·44 | 99·15 | −0·29 | −ve |

The mean tumour temperatures before and during therapy are shown in Table I, as are the assessments of response. For only 10 of our patients has sufficient time elapsed to permit unequivocal assessment, but the results do indicate that a rise in tumour temperature appears to be related to a subsequent response, while a fall suggests that the patient will not benefit from the treatment.

Because of the small numbers in our series to date the statistical analysis used was an exact probability determination based on a 2 × 2 contingency table. The probability of obtaining the observed results was found significant at the 1% level ($P = 0{\cdot}0048$).

The fact that such a simple single parameter provides an indication of subsequent response is extremely encouraging and it is hoped that more sophisticated analysis using some combination of several parameters as a discriminant function will prove to be even more definitive, perhaps enabling the degree or duration of response to be predicted.

Inspection of the traces has revealed several other interesting features:

1. In all patients the tumour temperature was at all times greater than that of normal breast tissue (Fig. 1).
2. All probes implanted peripherally in the tumour showed very similar results (Fig. 2). We suspect that the central region of a large tumour may be colder than its periphery, but have not performed sufficient central measurements to confirm this.
3. The difference in temperature between tumour and normal tissue in any one patient varied considerably throughout any 24-hour period. The maximum difference varied from 1·14° F. in one patient to 8·14° F. in another. This variation is clearly shown in Fig. 1 where at 38 hours the

FIG. 1.—Recording illustrates tumour temperature is at all times higher than that of normal breast tissue; this was typical of all patients in the series.

FIG. 2.—Typical recording illustrating that the three independent probes implanted in the tumour exhibited similar temperatures.

difference between tumour and normal tissue is 2·3° F. and approximately 6 hours later has reduced to 0·25° F.

4. All patients exhibited a cyclic temperature variation both in tumour and in normal breast both before and during therapy (Fig. 3). The period of this cycling was approximately 24 hours in all cases and the cycles in tumour and normal breast were in phase. On visual inspection no correlation has yet been observed between either period or amplitude and subsequent response.
5. Temperature changes in normal breast tissue appear to be unrelated to response.
6. Skin temperature measurements directly over the tumour in some cases do, and in other do not, correlate with the results of the implanted probes. Only in cases where the tumour actually involves the skin are the results comparable (Fig. 4).

FIG. 3.—In all patients tested a circadian rhythm was observed in both tumour and normal tissue, both before and during therapy. In the traces shown, short term fluctuations have been smoothed by applying a 10 term moving average technique, this method has proved of value in enabling circadian rhythm to be directly recognized.

FIG. 4(a).—Tumour did not involve the skin. The surface probe placed directly above does not accurately reflect tumour temperature (smoothed data).

FIG. 4(b).—Tumour involved the skin. In this case the implanted probes and the surface probe gave similar results. (This trace is the smoothed version of that shown in Fig. 2 and illustrates the effect of the smoothing technique.)

### DISCUSSION

There have been several attempts to enable the effectiveness of therapeutic agents to be predicted.

In patients suffering from cancer of the uterine cervix, Graham (1947), Graham and Graham (1956) and Gilmore and co-workers (1949) attempted to select patients suitable for surgery rather than radiation therapy on the basis of cell changes before and after trial irradiation.

Radioisotope methods have been explored by Hale (1961) and Bullen *et al.* (1963) using phosphorus-32. All these workers proposed that variations in the $^{32}P$ uptake pattern in tumour induced by hormone administration might reflect changes in tumour activity.

Edelstyn and co-workers (1968) proposed certain methods of selecting patients likely to benefit from hypophysectomy.

All these methods leave much uncertainty and none have gained general acceptance.

Lloyd-Williams (1964) investigated a small number of patients with breast cancer taking isolated thermographic recordings at daily intervals and found that in certain instances hormone administration produced a rise in tumour temperature whilst in other instances a fall was noted. Where temperature fell clinical regression occurred, and when it rose the disease seemed to be exacerbated. He does, however, state that environmental conditions were open to criticism.

Mansfield *et al.* (1968) developed this technique further, carrying out continuous monitoring of temperature over a period of some 10 to 14 days using thermistor probes fixed to the skin. His principal finding was that in patients subsequently showing a favourable response the most important change in the tumour temperature pattern was production or accentuation of a normal circadian rhythm. A fall in tumour temperature, as observed by Lloyd-Williams (1964) was also noted in this group. Our results suggest that measurements taken from the skin overlying the tumour may not reflect tumour temperature changes as accurately as implanted probes. Dodd and co-workers (1969) suggested that there may be no direct relationship between the area of increased heat emission and the location of the tumour, and that not all cancers produce enough heat to result in surface temperature gradients sufficiently pronounced to give a positive thermograph. Unless there is skin involvement the transfer of heat from tumour to skin surface is mainly by venous convection, direct conduction is probably a small factor due to the excellent insulating properties of body fat.

The technique we adopted could be criticized on the grounds that trauma will be caused by implanting the probes and that different probe positions within the tumour might give differing results due to the small volume of tissue being monitored and that the impossibility of implanting every probe in the same relation to neighbouring vascular structure. However, the small dimensions of the probes and the implantation technique adopted should minimize trauma, and results from multiple probes being implanted in the same tumour have shown that, provided the central tumour region is avoided, reasonably consistent temperatures and patterns will be obtained.

Our finding that marked variations occur in the tumour–normal differential within short times casts considerable doubt on the value of the " normal " thermogram. The thermogram seems to be gaining wide acceptance as a method of screening for breast cancer and involves taking an isolated measurement on the surface temperature pattern over the breasts. Our data would further suggest that the correlation between degree of tumour temperature elevation and prognosis, proposed by Lawson and Gaston (1964) and Lloyd-Williams (1964), could be stronger if peak values of elevation were used rather than isolated measurements at an unknown position on the temperature cycle. The results of Mansfield *et al.*, (1968) and of Lloyd-Williams also exhibited this varying differential.

It is of interest that the temperature changes observed in our patients were contradictory to those of Mansfield and Lloyd-Williams. All patients failing to respond exhibited a slight but definite fall in mean tumour temperature on preliminary hormone administration, whilst all responders showed a rise. At this stage we would neither attempt to explain these results nor to draw very firm conclusions; however, the subject clearly requires further investigation under rigorously controlled conditions and as the number of patients in our series increases we hope soon to be in a position to make a more confident assessment of this technique.

It is a pleasure to express our gratitude to Messrs. Schering A. G., Berlin, for the generous financial assistance without which this work could not have been undertaken, and especially to their Medical Advisers, Doctors Pitchford and Bye. Our research assistant Mr. A. McAteer deserves special mention as does the Northern Ireland Hospitals Authority who provided financial support for his services. We are also grateful to the Sister and Nursing staff of Ward 5C, Theatre Sister, and to Mrs. Layland for typing.

### REFERENCES

BULLEN, M. A., FREUNDLICH, H. F., HALE, B. T., MARSHALL, D. H. AND TUDWAY, R. C. —(1963) *Post-grad. med. J.*, **39**, 265.

DODD, G. D., WALLACE, J. D., FREUNDLICH, I. M., MARSH, L. AND ZERMINO, A.—(1969) *Cancer, N.Y.*, **23**, 797.

EDELSTYN, G. A., GLEDHILL, C. A. AND LYONS, A. R.—(1968) *Clin. Radiol.*, **19**, 426.

GILMORE, M., GLUCKSMANN, A. AND SPEAR, F. G.—(1949) *Br. J. Radiol.*, **22**, 90.

GRAHAM, J. B. AND GRAHAM, R. M.—(1956) *Ann. New York Acad. Sci.*, **63**, 1458.

GRAHAM, R. M.—(1947) *Surgery Gynec. Obstet.*, **84**, 153.

HALE, B. T.—(1961) *Lancet*, ii, 345.

LAWSON, R. N. AND GASTON, J. P.—(1964) *Ann. New York Acad. Sci.*, **121**, 90.

LLOYD-WILLIAMS, K.—(1964) *Ann. New York Acad. Sci.*, **121**, 272.

MANSFIELD, C. M., DODD, G. D., WALLACE, J. D., KRAMER, S. AND CURLEY, R. F.—(1968) *Radiology*, **91**, 673.

*Note added in proof:* Results up to March 1971 are as follows: In 18 out of 22 patients an increase in tumour temperature on SH420 administration has implied a subsequent response and a decrease a lack of response. In the two patients whose tumour temperature fell, but who responded, a marked reduction in the amplitude of circadian rhythm was noted.